# New carbon/ZnO/Li_2_O nanocomposites with enhanced photocatalytic activity

**DOI:** 10.1038/s41598-019-53335-7

**Published:** 2019-11-14

**Authors:** Aurel Diacon, Alexandra Mocanu, Cristian Eugen Răducanu, Cristina Busuioc, Raluca Șomoghi, Bogdan Trică, Adrian Dinescu, Edina Rusen

**Affiliations:** 1 University POLITEHNICA of Bucharest, Faculty of Applied Chemistry and Materials Science, Gh. Polizu Street 1-7, Bucharest, postal code 011061 Romania; 2 National Research and Development Institute for Chemistry and Petrochemistry – ICECHIM, 202 Splaiul Independenţei, Bucharest, 060021 Romania; 3 National Institute for Research and Development in Microtechnologies - IMT-Bucharest, 126 A, Erou Iancu Nicolae Street, PO-BOX 38-160, 023573, Bucharest, 077190 Romania

**Keywords:** Materials for energy and catalysis, Pollution remediation

## Abstract

Our study was focused on the synthesis of photocatalytic materials for the degradation of organic dyes based on the valorization of biomass resources. The biochar resulted from pyrolysis process of cherry pits wastes was activated by CO_2_ flow. Activated and inactivated carbon was used to obtain carbon-based photocatalysts impregnated with different zinc salt precursors. The activation of carbon had no significant influence on the photodegradation process. The doping procedure used Li_2_CO_3_ and Zn(CH_3_COO)_2_ of different concentrations to impregnate the biochar. The *carbon-ZnO-Li*_2_*O* based nanomaterials were analysed by TEM and SEM, while the presence of hexagonal wurtzite ZnO was investigated by XRD. The solid samples were analysed by PL at 360 nm excitation fixed wavelength to correlate their morphology with the optical and photocatalytic properties. The presence of Li atoms led to photocatalytic activities of the doped ZnO similar to the undoped ZnO obtained at higher concentrations of zinc acetate precursor.

## Introduction

Water contaminants and organic pollutants represent a tremendous problem even in the 21^st^ century leading to serious health issues in the population like dizziness, respiratory allergies, gastrointestinal pains, skin eczema, genetic malformations and even infertility^1^. Textile industry requires huge amounts of methyl orange (MO) as dye agent. Being rated as toxic compound with carcinogenic effects and extremely low biodegradability, MO can cause important long-term fauna and flora hazards^2,3^.

Commercial active carbon (CA) gained much interest in the field of wastewater decontamination due to its high specific surface, increased thermal stability, uniform porous morphology and reduced toxicity. Unfortunately, the manufacturing costs for active carbon as water purifying compound is relatively high since additional procedures for its chemical activation is needed^4^. The conversion of biomass into new products with added value can be achieved through different processes such as: combustion, torrefaction, liquefaction, gasification and pyrolysis^5^. According to European Directive 2008/98/EC, pyrolysis is considered a recovery operation and recycling technology as well as a technological method to obtain new products by reconversion of organic wastes (including biomass)^6,7^. For this reason, in the last decade, the research studies involved the use of bio-char obtained from pyrolysis of biomass such as almond or coconut shells, wood chips or different plant waste from agricultural industry^8,9^ due to its decontamination potential and disinfection characteristics which led to degradation of pesticides, organic dyes, pharmaceutical chemicals or the membrane cell wall of certain pathogen bacteria (*E. coli*, *S. aureus*, etc.)^10^.

The removal of organic dyes may involve several methods such as reverse osmosis, membrane filtration, ion exchange, reducing/oxidation or advanced oxidation processes. The treatment of wastewater by advanced oxidation process implied the use of inorganic metal oxides with intrinsic photocatalytic properties (i.e.: ZnO, SnO, TiO_2_, etc.)^11–15^ that generate hydroxyl free radicals as oxidizing agents that decompose the organic contaminants to CO_2_ and H_2_O in the presence of UV light, hydrogen peroxide or ozone^2,15–17^. Compared to pure inorganic metal oxides, the nanomaterials that use biochar as support for the deposition of the inorganic particles can promote the photodegradation of the organic dyes with increased degradation rate^18–20^. Thus, several research studies not only demonstrated higher efficiencies in terms of decomposition of the organic dyes, but also confirmed that the structure, morphology, or the functional groups present on the surface of the biochar are extremely important for the immobilization and/or synthesis of the metal oxides nanoparticles leading to an enhanced photocatalytic degradation process^18,20,21^. Recent studies were also directed to carbon-semiconductor materials for photocatalytic applications which used graphene, graphene oxide or modified solvent exfoliated graphene as carbonaceous precursors^22^. By different “*in situ*” or “*ex situ*” techniques, semiconducting materials like CdS-TiO_2_, TiO_2_, or ZnO were generated to “decorate” carbon nanotubes or graphene sheets^23–25^. The “*ex situ*” method mainly involved the use of commercially available semiconductors or preformed photocatalytic nanostructures which were mixed with graphene sheets or precursors of graphene. In contrast, the “*in situ*” procedure used solutions of semiconductor precursors mixed with the precursors of graphene. Although the “*ex situ*” synthesis method is less efficient in terms of interfacial interaction between the semiconducting nanostructures and graphene surface, this procedure offered a better control over the morphology of photocatalytic semiconductors^22^.

Based on these synthesis procedures, one of our aims was to use an “*in situ*” synthesis method with the advantages of an “*ex situ*” technique. Therefore, our approach involved the use of several zinc salts aqueous solutions to impregnate the biochar structure followed by solvent removal and calcination of the biochar and generation of semiconductor materials in order to investigate the effects on the morphology of the obtained ZnO and its photocatalytic activity.

Although metal oxides like TiO_2_ and ZnO are successfully used in photocatalytic degradation of organic compounds due to their semiconducting properties, thermal stability, low-toxicity, high natural abundance and reduced producing costs, a lot of efforts are being made to design different materials to improve their photocatalytic activity also in the visible range^26^ by certain techniques like noble metal deposition, doping procedures, modulation of vacancies in the semiconducting material, use of carbonaceous materials or quantum dots^27^.

In recent studies, carbon materials like graphene or carbon nanotubes were doped with semiconducting materials (TiO_2_ nanostructures arrays) and nanocrystalline particles (Au, Pd, Sb_2_S_3_, etc.) for an electron flow from semiconductor to metal and to prevent the recombination of the photoexcited electron-hole pairs by a cascade charge transfer photocatalytic mechanism in photoelectrochemical cells^28–31^. Electron-hole pairs formation (**e**^**−**^ − **h**^**+**^) are the result of light irradiation of carbon-semiconductor-metal. The electrons from the conduction band of metal nanoparticles pass to the conduction band of the semiconductor inducing an energy level alignment between the two materials. Simultaneously, the holes from the valance band of semiconductor will flow to the valance band of the metal, thus promoting the separation of **e**^**−**^ − **h**^**+**^ charge carriers. The carbonaceous material transports the electrons accumulated in the conduction band of the semiconductor promoting directional electron transport. Furthermore, the holes from the valence band of the metal nanoparticles enable water oxidation forming oxygen active species that prevent the recombination of photoinduced **e**^**−**^ − **h**^**+**^ pairs thus prolonging the lifetime of the charge carriers^31^.

Doping agents like copper, yttrium, silver, cerium, etc. ranging from 1 to 5% in weight were used to decrease the energy band-gap of ZnO or TiO_2_^17,26,32–34^ in order to create oxygen vacancies and prevent the recombination of electron-hole (**e**^**−**^ − **h**^**+**^) pairs, thus increasing their photocatalytic activity also in visible light. Depending on the synthesis method, in some cases, the dopant had an inhibiting effect on the crystallite growth of the semiconducting material (ZnO/TiO_2_), thus leading to an increased specific area of the final photocatalyst which registered enhanced efficiencies during the photodegradation process^32,35–38^.

Until now, few studies demonstrated that the use of Li_2_O as dopant may enhance the performance of certain metal oxides like Co_3_O_4_, Mn_2_O_3_, CuO, ZnO, etc. in terms of catalytic activity^39,40^ or sensitivity^41^ due to the morphologic modifications that appear during the synthesis process which led to increased specific surface area of the inorganic final materials.

Thus, the aim of our study is to investigate the role of biomass carbon as support on one hand, and the role of Li_2_O as dopant (using Li_2_CO_3_ with concentrations of 0.05 M, 0.1 M, and 0.2 M) on the other, for ZnO-based photocatalytic structures for the decomposition of MO. Furthermore, our research study involved also the use of different zinc salt precursors for ZnO structures and the influence of their concentration in the composite synthesis in the photodegradation process. Using Li_2_O as dopant in the carbon-ZnO based composites for photocatalytic degradation of organic dyes represents a premier until now to our knowledge.

## Characterization

The micrographs for the carbon/ZnO/Li2O photocatalysts were obtained by scanning electron microscopy (SEM) using FEGSEM-Nova NanoSEM 630 (FEI).

The crystallographic planes of inorganic ZnO were analysed using x-ray powder diffraction (XRD) performed on a Panalytical X’Pert MRD system (λ CuKα = 0.15418 nm radiation). The spectra were recorded between 20 and 70°.

The detailed structures of our samples were analyzed by FEI Tecnai F20 G2 TWIN TEM equipped with EDX XMaxN 80 T detector from Oxford Instruments. The samples were first dispersed in absolute ethanol, deposited on copper grids and dried. The images were obtained at 200 kV, while the gun was connected to a high voltage source.

The values for the specific surface areas of the composite photocatalysts were determined with a gas (N_2_) porosimeter type Gemini V and on the basis of BET method.

The photodegradation monitoring of the experiment involved the decomposition of 500 mL MO with a concentration of 5 · 10^−5^ M at pH = 6 using a modified commercial set-up described elsewhere^15^. The set-up was comprised of a microdosing pump Masterflex-Unipan 335 with a flow fixed at 350 mL/h; a UV light bulb tube was introduced inside the quartz tube (𝜆 = 360 nm, power 4 W); a 5 mL flask was used to collect samples. Before the start of the experiment, the samples were kept in the dark for 30 min.

The degradation of MO was analysed by UV-VIS absorption. The spectra were recorded with an Able Jasco V-550 spectrophotometer; for the solid samples, reflection spectra were registered using an integrating sphere at normal incidence, a band width of 1 nm, and a scanning speed of 1000 nm min^−1^.

The fluorescence spectra have been registered using a FP-6500 Able Jasco spectrofluorometer at 360 nm excitation fixed wavelength for all composite samples.

## Results and Discussions

In the first step of our study, inactivated carbon (CN) was obtained by pyrolysis process of cherry pits (as described in the Methods section of the manuscript), while activated carbon (CA) was obtained by keeping the previously pyrolyzed cherry pits at 1200 °C using CO_2_ as flow gas for 1 hour.

One of our major objectives was to investigate the influence of activated carbon (CA) and inactivated carbon (CN) in the synthesis process of different ZnO structures that could further influence the photodegradation process of organic dyes.

**Figure 1:**
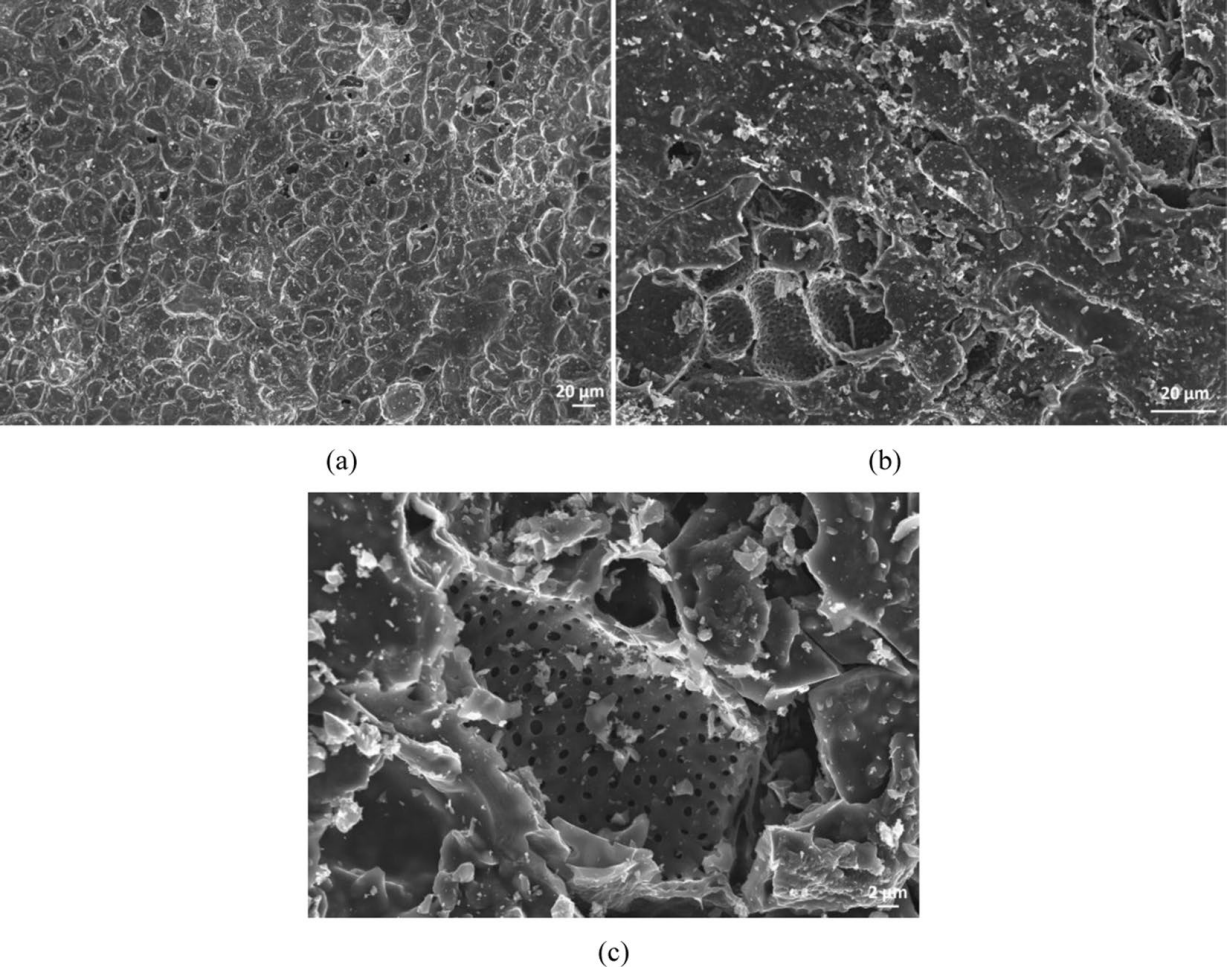
SEM micrographs for pyrolyzed cherry pits: (**a**) inactivated carbon (CN), (**b**) activated carbon (CA), (**c**) detail image of CA.

In our case, our first step consisted in the investigation of the morphologic structures of the carbon resulted from the pyrolyzed cherry pits (Fig. 1a), respectively activated carbon (Fig. 1b,c). The SEM images revealed that CN presents compact surface with few porous areas (Fig. 1a), while CA indicates an increased specific surface due to a higher density of uniform holes/pores on the surface (Fig. 1c - detail image).

The next step involved the use of both CN and CA biochar as support to generate ZnO by the impregnation of the black powder with 3 g solution of zinc acetate precursor, followed by evaporation and calcination (as described in *Section* 2*.2*).

**Figure 2:**
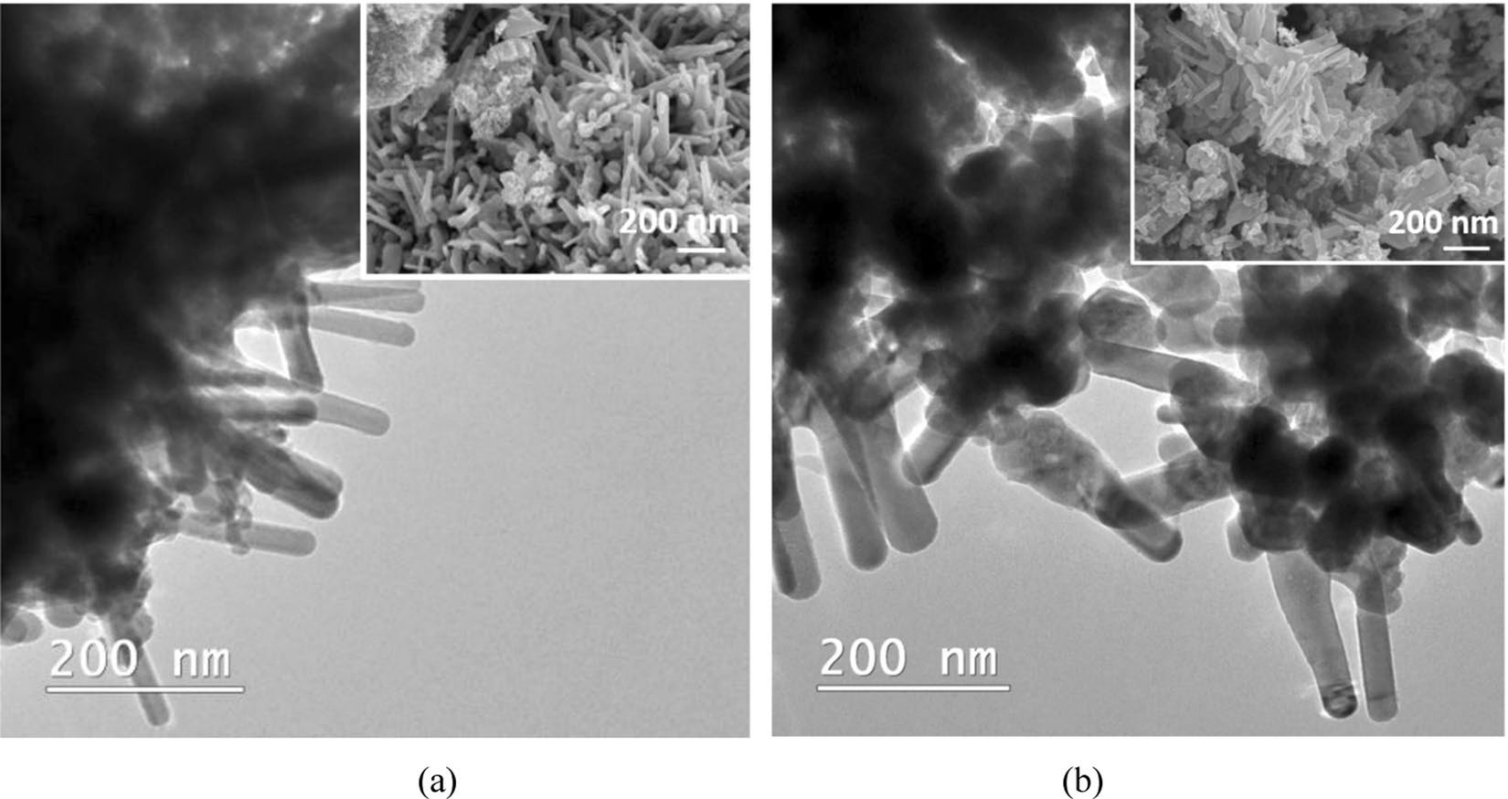
TEM image and SEM micrographs (as details) of (**a**) CN-ZnO-3 and (**b**) CA-ZnO-3 after calcination at 500 °C for 4 h.

The TEM images in Fig. 2 obtained for both carbon-zinc acetate-based composites after calcination did not revealed strong differences in terms of morphology. Both samples represent a mixture of agglomerated nanoparticles, relatively long rods or bowling pins structures with hexagonal cross-section (at least 250 nm in length and almost 40 nm in diameter). The SEM micrographs insets provide also an overview of both samples confirming the similarities of the ZnO shapes formed in the presence of CN (Fig. 2a), respectively CA (Fig. 2b).

**Figure 3:**
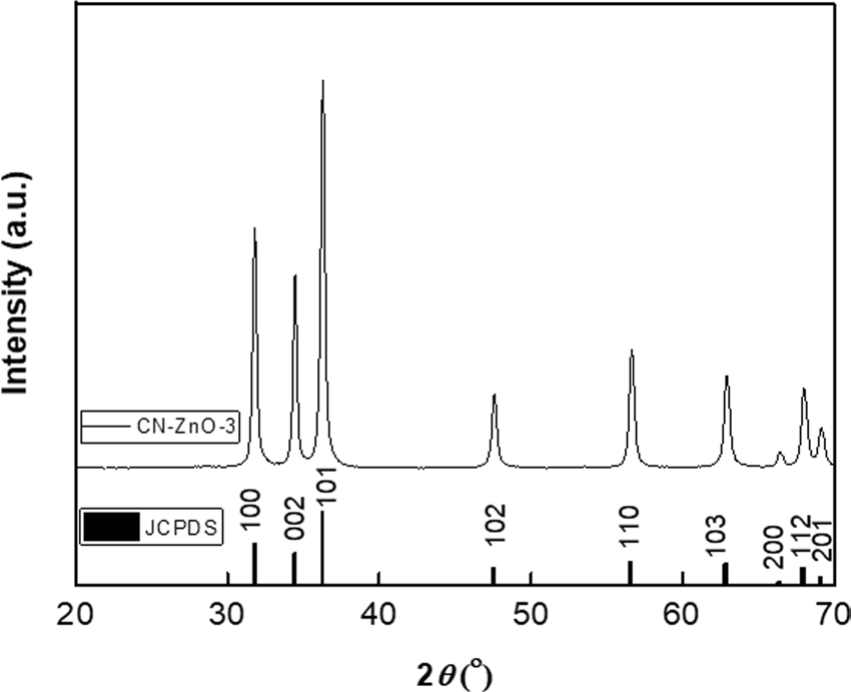
XRD analysis of carbon-zinc acetate-based nanocomposites after calcination.

In order to confirm the presence of ZnO structures after the calcination procedure, XRD analyses was performed (Fig. 3) and the spectra were identical for both samples (CA-ZnO-3, respectively CN-ZnO-3).

The diffraction peaks registered at 2θ = 31.74°, 34.48°, 36.23°, 47.55°, 56.69°, 62.93°, 66.56°, 68.01°, 69.16° correspond to (100), (002), (101), (102), (110), (103), (200), (112), respectively (201) crystallographic planes of wurtzite hexagonal structure of ZnO according to JCPDS no. 036–1451.

Consequently, 0.4 g of each nanocomposite was used to photocatalytic decompose the (MO) solution of 5·10^−5^ M. The process was monitored for 6 hours and the absorption spectra of the extracted samples were recorded using UV-Vis analysis. The performance of the photocatalysts was estimated in terms of efficiency:

$${\rm{Efficiency}}=\frac{Ab{s}_{0}-Ab{s}_{n}}{Ab{s}_{0}}\cdot 100$$

where, **Abs**_**0**_ represents the initial absorbance of the MO solution, while **Abs**_**n**_ represents the absorbance of MO registered every hour.

**Figure 4:**
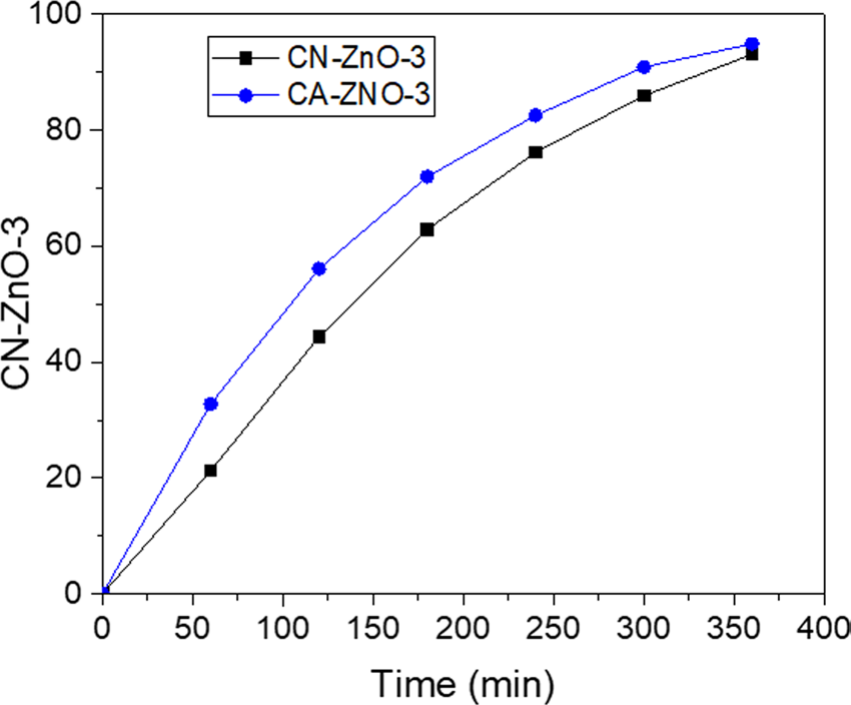
Efficiency of the photodegradation process of MO solution (5·10^−5^ M) using: (**a**) CA-ZnO-3, respectively (**b**) CN-ZnO-3 photocatalysts.

As expected, the photodegradation process of MO indicated that the differences between the two photocatalysts are slightly different (Fig. 4). In our case, the results for efficiency after 6 hours were almost the same (94.8% for CA-ZnO-3, respectively 93% for CN-ZnO-3), thus concluding that in this case, the activation of carbon powder has a small amplification effect on the photodegradation process of MO (Fig. 4).

Considering that the manufacturing procedures of our materials must involve as few processing steps as possible and that the activation of carbon had minor effect on the photocatalytic degradation of MO, we focused on the optimization of carbon-ZnO based photocatalysts using only non-activated carbon as support for the synthesis of the inorganic photocatalytic structures.

The next step of this study was to investigate the efficiency of the photocatalytic materials using different salt precursors for the generation of ZnO structures. Thus, we used the same concentration for ZnCl_2_, respectively Zn(NO_3_)_2_ · 6H_2_O solution (0.65 M) in order to impregnate the inactivated carbon powder. After calcination procedure, the CN-ZnO-Cl and CN-ZnO-NO_3_ white powders (0.4 g) were used to decompose MO (5 · 10^−5^ M solution).

**Figure 5:**
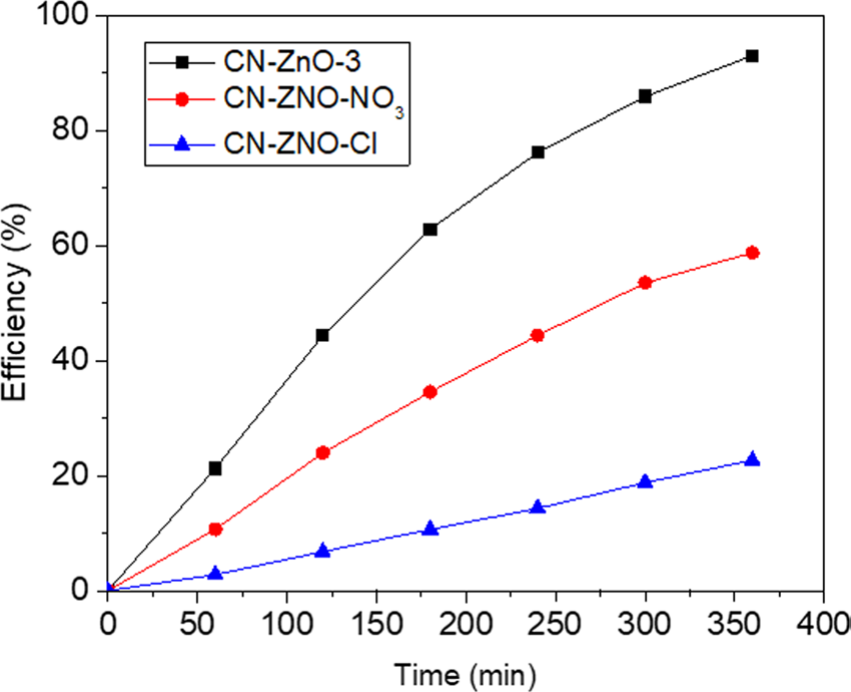
Efficiency of ZnO salt precursors in the photodegradation process of MO (5 · 10^−5^ M).

**Figure 6:**
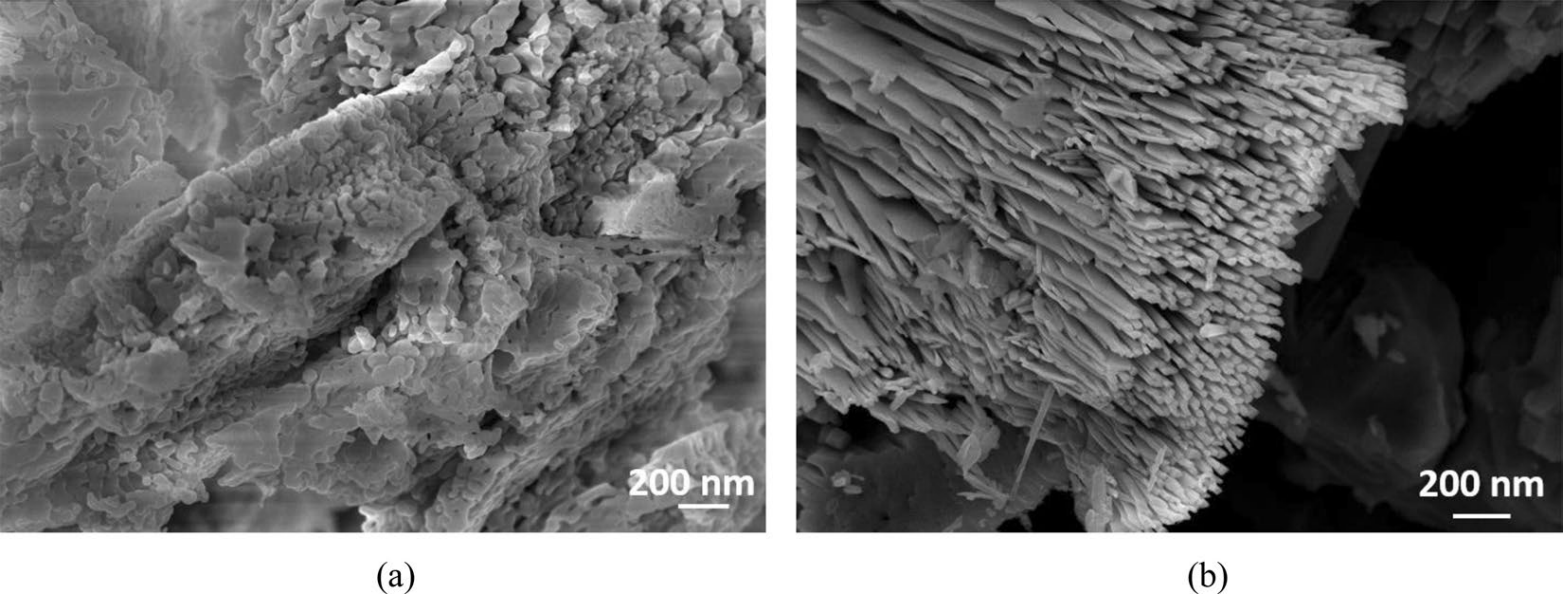
SEM images of carbon-ZnO-based nanocomposites using different salt precursor: (**a**) CN-ZnO-NO_3_, and (**b**) CN-ZnO-Cl.

The efficiency of the nanocomposites registered interesting values ranging from 22.7% for CN-ZnO-Cl_,_ respectively 58.8% for CN-ZnO-NO_3_ compared to 93% of CN-ZnO-3 (Fig. 5). These results could be related to the calcination procedure in the presence of oxygen atmosphere that occurs by different mechanisms depending of the zinc salt precursor^18,42^. Thus, a two-step process could explain the differences obtained in the morphologies of our composites (Fig. 6). In the case of zinc acetate precursor the first step represents a decomposition to CO, CO_2_, H_2_ and water which inhibit the oxidation of Zn, while the second step implies the crystalline growth of rod-like structures based on the ZnO nanoparticles formed in the first step on the surface of the support^42^. In our case, Fig. 6 presents the different morphologies of the composites resulted from the use of chloride, respectively nitrate zinc salt proving that the decomposition step occurs without inhibition growth of the ZnO structures. As a consequence, the ZnO structures obtained from CN-ZnO-NO_3_ appeared as agglomerates of fused particles (Fig. 6a) explaining the smaller efficiency in the photocatalytic degradation of MO compared to zinc acetate precursor (Fig. 2a)^43^. In Fig. 6b, the SEM image showed that CN-ZnO-Cl structures were disposed as cross-section hexagonal agglomerated brush-like nanorods or plate-like structures which probably led to the decrease of specific surface confirming the lower values of the efficiency for MO degradation.

**Figure 7:**
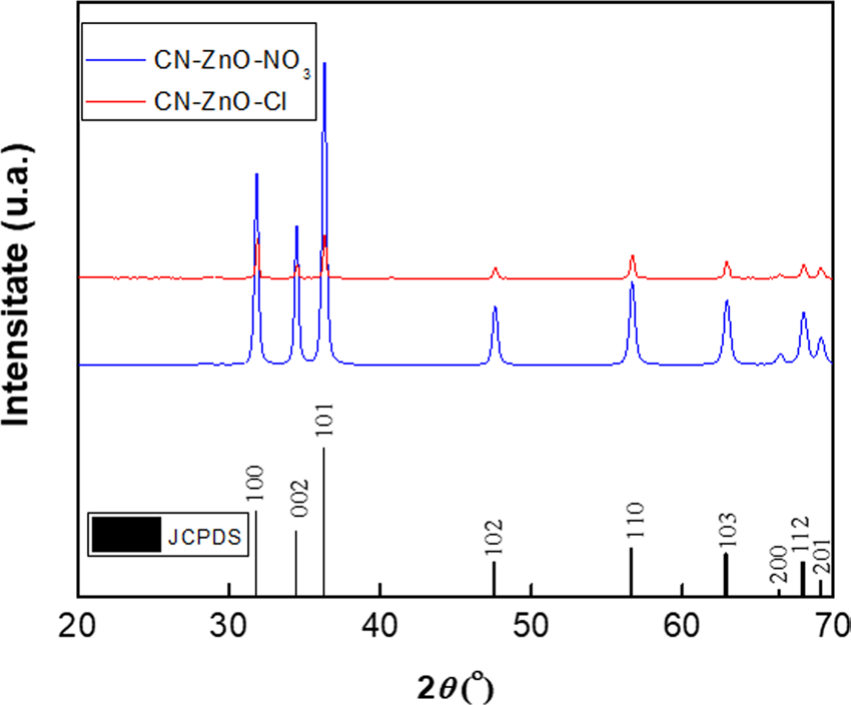
XRD spectra of carbon-ZnO-based nanocomposites using different salt precursor: CN-ZnO-NO_3_ – blue line, respectively CN-ZnO-Cl – red line.

These structural differences between the CN-ZnO-Cl and CN-ZnO-NO3 were also confirmed by XRD spectra. Although the diffraction peaks identified the wurtzite hexagonal ZnO phase in both cases (JCPDS no. 036-1451 ASTM file), the intensity peaks of the photocatalyst resulted from the use of zinc nitrate are considerably higher due to increased crystallinity of the sample (Fig. 7)^44^. Thus, these results are in good accordance with the photodegradation process of MO. It is worth mentioning that no impurity peaks were registered in Fig. 7 as the biochar support was either completely removed or in very low concentration after the calcination process for both samples, thus confirming literature available information^45^.

**Figure 8:**
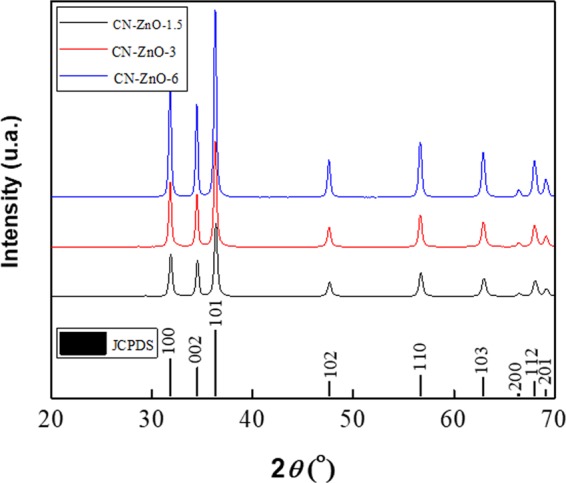
XRD spectra of photocatalysts obtained by increasing the amount of zinc acetate: CN-ZnO-1.5 (black like), CN-ZnO-3 (red line), respectively CN-ZnO-6 (blue line).

Based on these last results, our purpose was to improve the efficiency of our photocatalysts. Thus, the next step involved the study of photocatalytic activity of the samples based on increased amounts of zinc acetate. The inactivated biochar was impregnated with different amounts of zinc acetate 1.5, 3 and 6 g (*see* Table 1) and calcinated following the same conditions previously mentioned in the manuscript. The XRD presented in Fig. 8 for CN-ZnO-1.5, CN-ZnO-3, respectively CN-ZnO-6 materials, registered the diffraction peaks corresponding to hexagonal wurtzite ZnO structure (according to JCPDS no. 036-1451 ASTM file) in all cases. No impurity peaks were evidenced on the spectra, only higher intensity of the signals as the amount of zinc acetate was increased.

**Table 1:** Sample codes and synthesis conditions for ZnO, respectively ZnO-Li_2_O photocatalysts.

Sample code	Biochar	Biochar quantity (g)	Water (mL)	Zinc salt/Lithium salt precursor	Amount of zinc salt (g)	Amount of Li_2_CO_3_ g)
CA-ZnO-3	Activated biochar	1.1	50	Zn(CH_3_COO)_2_	3	—
CN-ZnO-1.5	Inactivated biochar	1.1	50	Zn(CH_3_COO)_2_	1.5	—
CN-ZnO-3	Inactivated biochar	1.1	50	Zn(CH_3_COO)_2_	3	—
CN-ZnO-6	Inactivated biochar	1.1	50	Zn(CH_3_COO)_2_	6	—
CN-ZnO-Cl	Inactivated biochar	1.1	50	ZnCl_2_	4.4	—
CN-ZnO-NO_3_	Inactivated biochar	1.1	50	Zn(NO_3_)_2_	9.6	—
CN-ZnO-Li_2_O-0.075	Inactivated biochar	1.1	50	Zn(CH_3_COO)_2_ /Li_2_CO_3_	3	0.075
CN-ZnO-Li_2_O-0.15	Inactivated biochar	1.1	50	Zn(CH_3_COO)_2_ /Li_2_CO_3_	3	0.15
CN-ZnO-Li_2_O-0.3	Inactivated biochar	1.1	50	Zn(CH_3_COO)_2_ /Li_2_CO_3_	3	0.3

**Figure 9:**
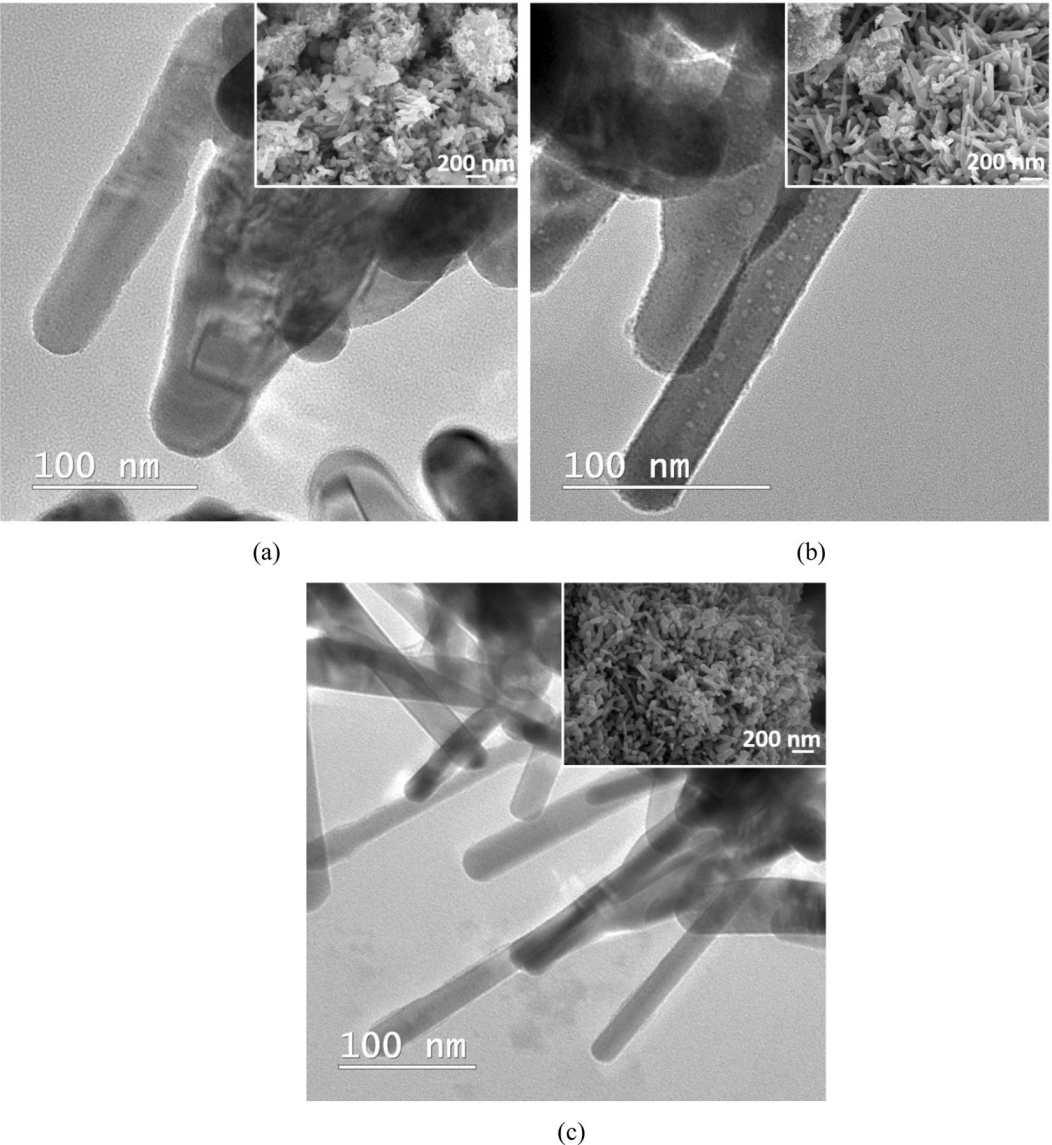
SEM image of (**a**) CN-ZnO-1.5, (**b**) CN-ZnO-3, and (**c**) CN-ZnO-6.

The ZnO structures are dominated by two types of aggregations in which ZnO nanoparticles bundle together and hexagonal rods or bowling pins are not clearly formed (Fig. 9a). As the amount of zinc acetate was increased, the CN-ZnO-3 registered ZnO rods with similar shapes as CN-ZnO-1.5, but the density of agglomerated nanoparticles was lower (Fig. 9b). At the highest concentration of Zn(CH_3_COO)_2_, ZnO structures have a more homogeneous aspect, being predominantly represented by well-defined hexagonal shaped rods and individual smaller nanospheres (Fig. 9c).

**Figure 10:**
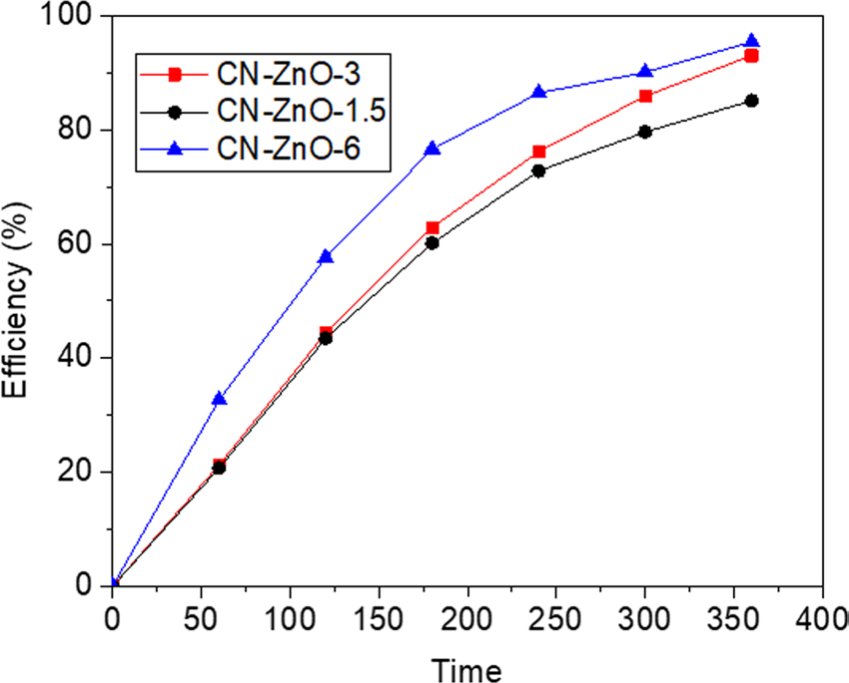
Efficiency results of the photocatalysts with higher amounts of Zn(CH_3_COO)_2_ for the photodegradation of MO (5 · 10^−5^ M): CN-ZnO-1.5 (black line), CN-ZnO-3 (red line), CN-ZnO-6 (blue line)

As expected, the efficiency values raised as the amount of Zn(CH_3_COO)_2_ was increased (Fig. 10) which is good correspondence with literature data as well^46^. The photodegradation process of MO using CN-ZnO-6 is faster and registered 86.4% efficiency after 4 hours (95.4% after 6 hours), while the efficiency of CN-ZnO-3 was 10% lower. The efficiencies of CN-ZnO-3 (93%) and CN-ZnO-1.5 (85.2%) were almost identical for the first 180 min (red and black lines), but had approximately 8% difference at the end of the experiment.

**Figure 11:**
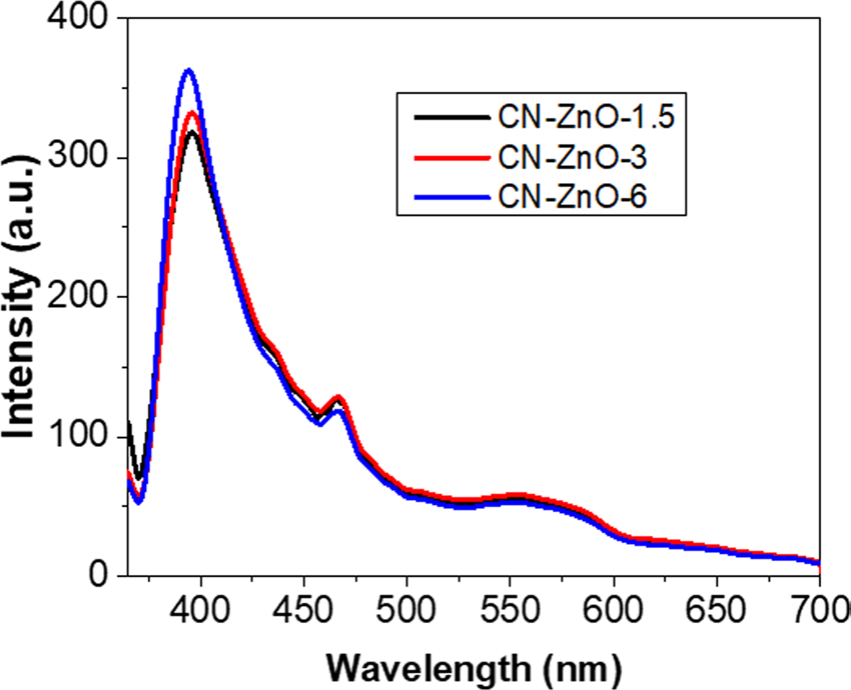
PL spectra registered at 360 nm excitation wavelength for CN-ZnO-1.5, CN-ZnO-3, and CN-ZnO-6 photocatalysts.

**Figure 12:**
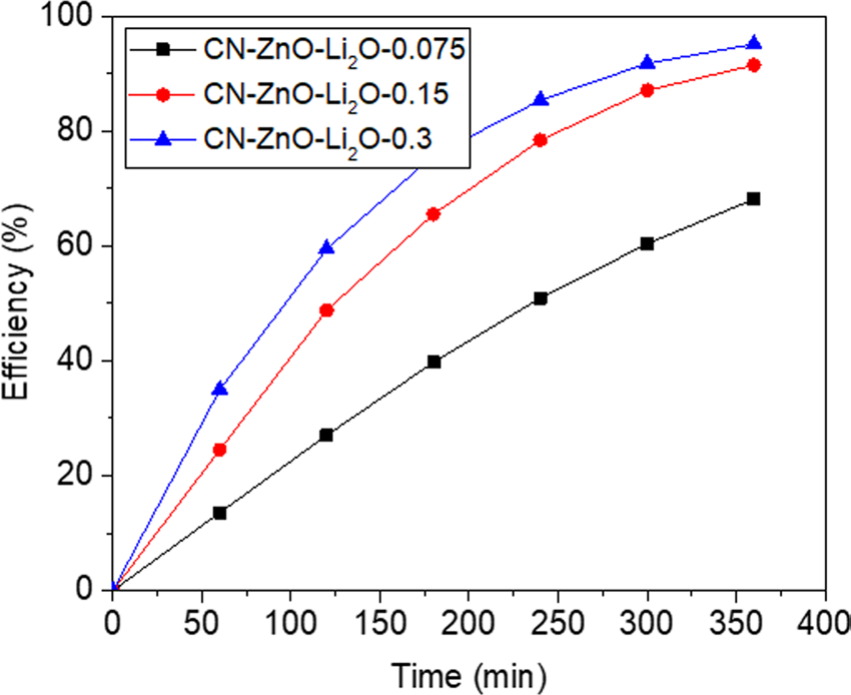
Graphic of efficiency-time for carbon-ZnO-Li_2_O based photocatalyst for degradation of MO (5 · 10^−5^ M).

Considering these results, the PL of the samples was recorded at 360 nm fixed excitation wavelength (Fig. 11). The intensity of the spectra for CN-ZnO-3 (red line) is slightly higher compared to CN-ZnO-1.5 (black line) thus, confirming the differences in the morphologies of these two samples (Fig. 9a,b). The emission wavelengths of these samples were registered at 395 nm, thus proving the similar behavior in the photodegradation process (Fig. 10). Using the highest amount of Zn(CH_3_COO)_2_ in the synthesis of biochar-ZnO-based nanomaterials the highest PL intensity was registered for CN-ZnO-6 (Fig. 12-*blue line*) proving the results of the photodegradation of MO (Fig. 10) and the inorganic nanostructures showed by SEM micrographs (Fig. 9c).

Thus, at higher concentration of Zn(CH_3_COO)_2_, the intensity of the photoluminescence and the efficiencies of the photocatalytic activity of our materials were increasing.

Our next strategy of this study was to investigate the influence of Li as dopant for carbon-ZnO based materials in the photodegradation process. In some cases the use of doping agents to create defects in the emission wavelength spectra of ZnO proved high photocatalytic activities^17,47,48^, while in the case of Li the few studies that were carried out until now generated contentious opinions in terms of defect levels for p-type semiconductors manufacturing^49^.

In a typical experiment, Li_2_CO_3_ and Zn(CH_3_COO)_2_ solutions were simultaneously used to impregnate the inactivated carbon in different proportions. Aliquots of 3 g (0.65 M) Zn(CH_3_COO)_2_ and 0.075, 0.15, respectively 0.3 g of Li_2_CO_3_ were prepared to impregnate the inactivated carbon powder (according to Table 1). After water removal and calcination, the samples were used to decompose the MO (5·10^−5^ M) (Fig. 12).

**Figure 13:**
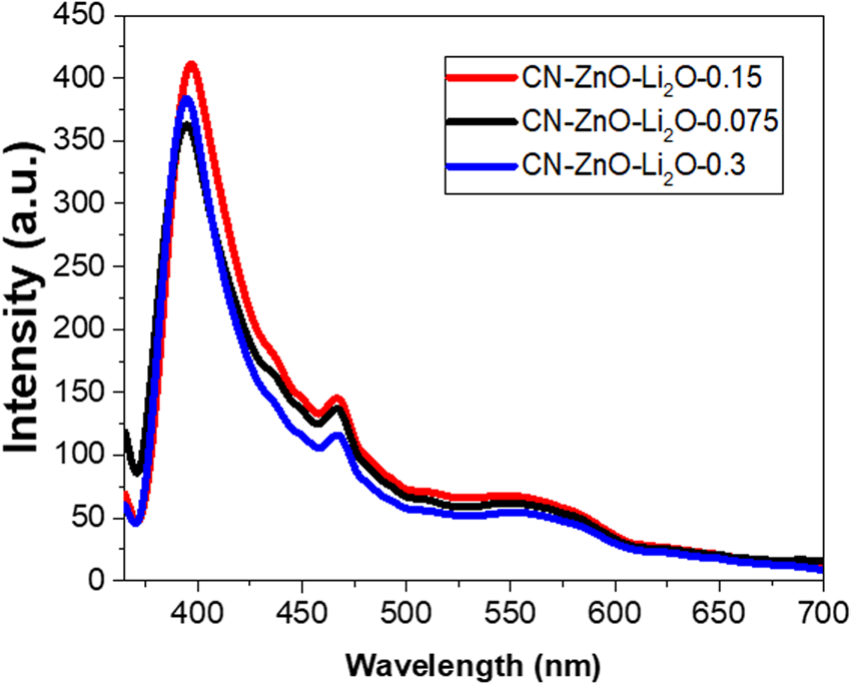
PL of carbon-based ZnO-Li_2_O photocatalysts at 360 nm excitation wavelength.

As expected, the efficiency increases as the amount of Li_2_CO_3_ was increased, registering values ranging from 68.2% to 97% after 6 h (Fig. 12). In order to characterize the samples doped with Li_2_O, PL spectra were registered at 360 nm fixed excitation wavelength (Fig. 13) and interesting results were obtained.

At higher concentration of Li_2_CO_3_ the photoluminescence of the samples was improved in the case of CN-ZnO-Li_2_O-0.075 and CN-ZnO-Li_2_O-0.15, but in the case of using the highest amount of Li precursor for CN-ZnO-Li_2_O-0.3 (blue line) a decrease in the intensity was observed compared to CN-ZnO-Li_2_O-0.15 (red line) (Fig. 13).

Two emission peaks were registered at 395 nm, respectively 466 nm in all cases. Compared to the undoped photocatalysts (Fig. 11) the intensity was slightly higher for the doped samples (Fig. 13). In our case, these peaks are registered at different values for both doped and undoped photocatalysts probably due to the synthesis method of our material and consequently to the morphology of our samples. Thus, the peak registered at 395 nm corresponds to the UV emission region (low near band edge – NBE)^49^ associated with the recombination of an electron and a hole in the valence band of ZnO, while the peak from 466 nm visible region is attributed to the transition of an electron from the conduction band to oxygen related defects in ZnO^50^.

In our case, the doping agent revealed some differences in the intensity of the PL spectra without noticeable shifts in the absorption band or defects in the ZnO band-gap, being also undetectable by XRD or EDS analysis. This results is in good agreement with literature data, Li atoms being hard to detect due to its close vicinity to the valence band of ZnO^49^.

**Figure 14:**
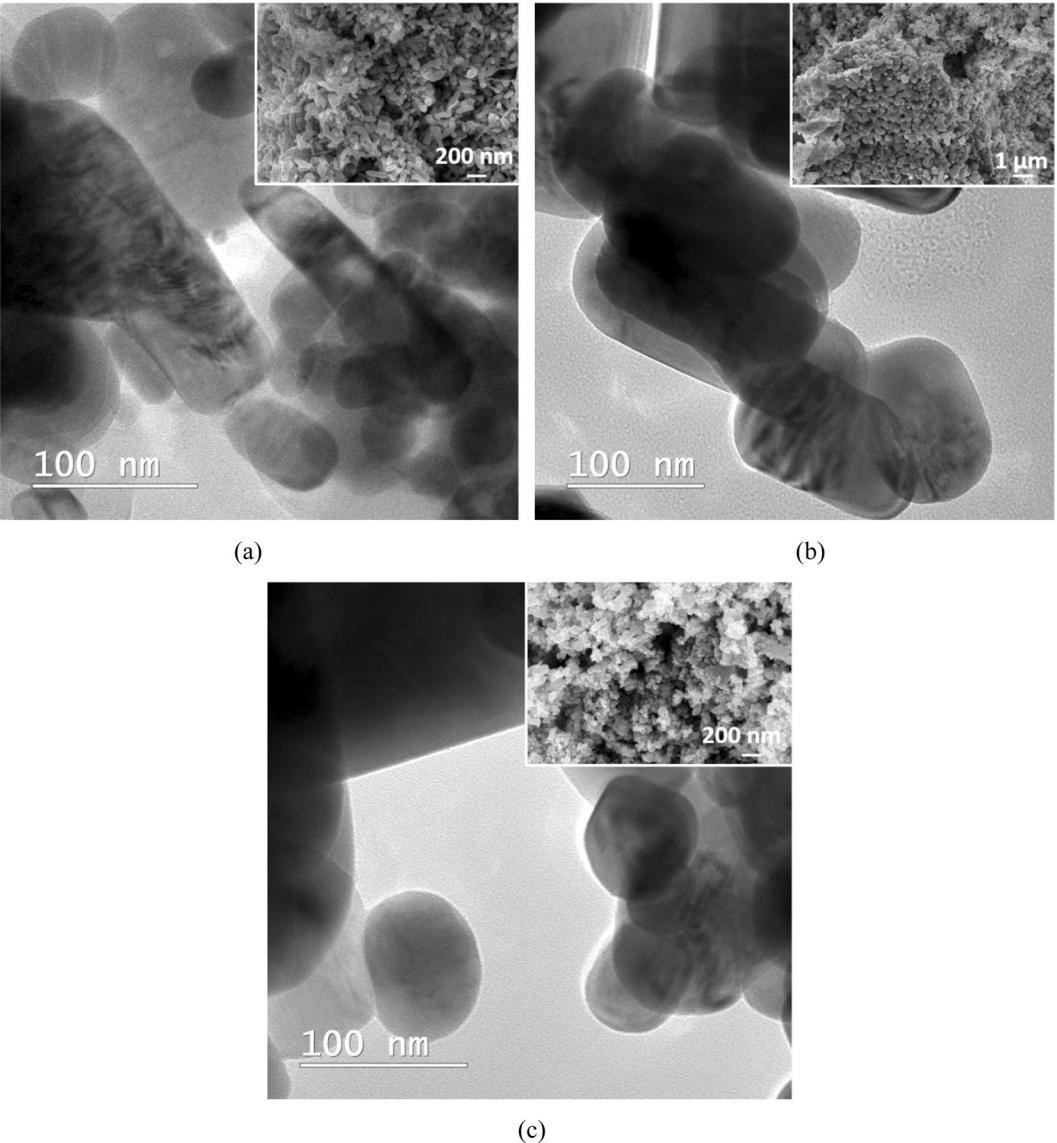
SEM image of (**a**) CN-ZnO-Li_2_O-0.075, (**b**) CN-ZnO-Li_2_O-0.15, (**c**) CN-ZnO-Li_2_O-0.3.

Nevertheless, the presence of Li atoms was observed in the photodegradation process of MO. The highest efficiency of the organic dye decomposition was registered in the case of using the highest concentration of Li_2_CO_3_ (Fig. 12), but this behaviour was not confirmed by the PL analysis, since CN-ZnO-Li_2_O-0.15 had the highest intensity of the emission spectra (Fig. 13). Consequently, the morphology of these samples was recorded by TEM and SEM analysis (Fig. 14) to indicate the differences in the morphology of our photocatalysts. Thus, as presented in Fig. 14, at increased amounts of doping agent, the morphology of the samples changed significantly from relatively polydisperse nanorods (Fig. 14a) to slightly monodisperse nanorods (Fig. 14b) and almost individual spherical shaped nanoparticles (Fig. 14c).

The photocatalytic properties of our samples can be connected with the band gap energies that were determined by UV-VIS reflectance analysis and estimated from Tauc equation^50^ (*plots are detailed in Supplementary Information* – Fig. S1, respectively S2). In Table 2, the band gap energy (Eg) slightly increases with the concentration of the doping agent. In certain cases greater values of band gaps indicate a decrease in the particles size^51^. In our case, the increase of the concentration of Li atoms induced an increase of the band gap and can also be correlated with a dramatic change in the morphology of the samples from polydisperse nanorods to almost spherical nanoparticles (Fig. 14). This is in good agreement with the photocatalytic activity of our samples that registered higher efficiencies for the smallest microstructures (Fig. 14c) in the degradation process of MO. Furthermore, although the doped samples did not register a significant change of the band gap values, the photocatalytic efficiency can also be attributed to an increase in the electron hole-pair mobility.

**Table 2:** The band gap energy values determined by Tauc plot (*detailed in Supplementary Information*).

Sample	Eg (eV)
CN-ZnO	3.22
CN-ZnO-Li_2_O-0.075	3.23
CN-ZnO-Li_2_O-0.15	3.24
CN-ZnO-Li_2_O-0.3	3.25

**Figure 15:**
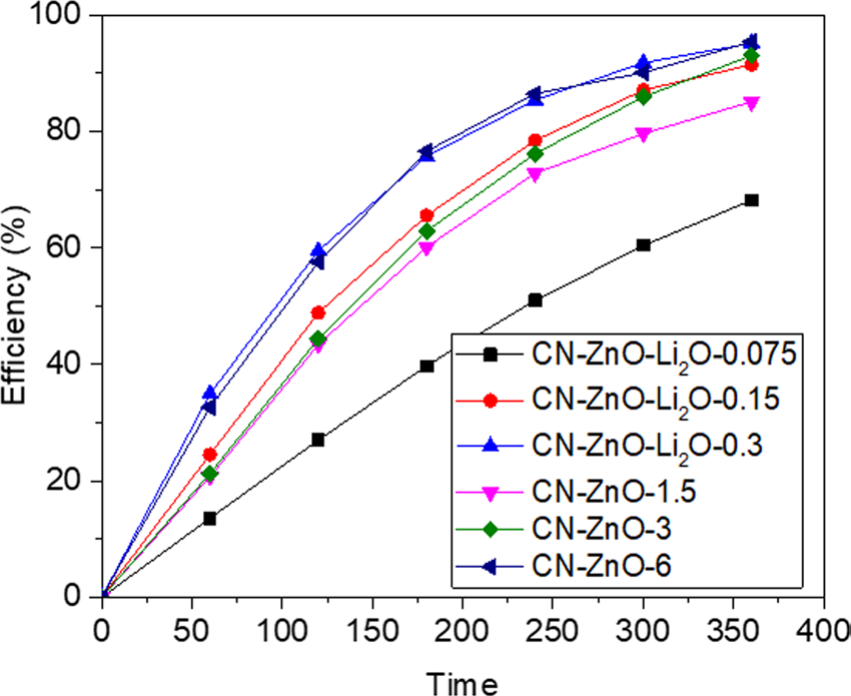
Efficiency-time photodegradation of doped and undoped photocatalysts.

Even more interesting results were obtained by comparing the photocatalytic activity of doped composites with unmodified ones (Fig. 15).

In Fig. 15, the presence of Li atoms led to a photocatalytic activity of the doped ZnO similar to the undoped ZnO obtained from zinc acetate precursor with higher concentration. Basically, CN-ZnO-Li_2_O-0.3 registered the same efficiency in the photodegradation process of MO as the undoped photocatalyst, CN-ZnO-6, in which 6 g of zinc acetate were used to obtained the carbon-inorganic based material. Comparable results were obtained for the pair CN-ZnO-Li_2_O-0.15 and CN-ZnO-0.3 that had almost identical efficiency values at the end of the photodegradation experiment. However, at very low concentration of Li atoms, the doping agent negatively influences the photocatalytic process since the efficiency registered for the decomposition of MO was less than 70% after 6 hours.

In order to establish a correlation between the photocatalytic activity of doped and undoped structures, BET surface was performed on CN-ZnO-3, CN-ZnO-Li_2_O-0.075, CN-ZnO-Li_2_O-0.15, and CN-ZnO-Li_2_O-0.3 (detailed in Table S1 - *Supplementary Information*). In the case of the samples doped with Li atoms a slight increase of the surface area was registered as the amount of dopant was increased (from 4.35 to 5.89 m^2^/g). This was also reflected in the photocatalytic activity of the doped samples (the efficiency of the composites increased with increasing the amount of doping agent). However, the BET surface area of the undoped sample CN-ZnO-3 registered a significantly higher value (10.88 m^2^/g). This difference can be explained by the synthesis procedure which led probably to a strong agglomeration^52–54^ of the ZnO and Li_2_O structures. Furthermore, there is no correlation between BET surface area and the photocatalytic activity since our samples which suggests that the most important parameter is the presence of the doping agent and electron-hole mobility modification^55^. The decrease of specific surface area while the photocatalytic activity increases in the presence of Li atoms can be also attributed to a better adsorption of the organic dye on the surface of the photocatalysts and the decrease in the recombination rate, since more defects are created in the structure of the semiconductor material (ZnO)^56^ at higher concentrations of doping agent.

In conclusion, we were able to evidence that at certain amounts of doping agent the photocatalytic activity of our carbon-ZnO-based materials was similar to those obtained with higher concentration of the zinc salt precursor.

Thus, this is a good premise to obtain less expensive photocatalysts using carbon from biomass wastes on one hand, and optimum concentrations of doping agent for the same photocatalytic activity as for undoped materials on the other.

## Conclusions

The carbon substrate was obtained by the pyrolysis process of cherry pits biomass wastes. The photocatalysts for the degradation of MO were obtained by applying an impregnation method of the activated or inactivated carbon using zinc salt precursors like zinc acetate, zinc nitrate or zinc chloride, followed by water removal and calcination.

The doping procedure was similar to the impregnation method, thus Li_2_CO_3_ and Zn(CH_3_COO)_2_ solutions were used to impregnate the biochar. The whole inorganic materials were analyzed by TEM and SEM to investigate the morphology of the samples, while the presence of hexagonal wurtzite ZnO was investigated by XRD. The photocatalytic decomposition of MO evidenced by UV-Vis absorption proved that the highest efficiencies of the degradation process were obtained in the case of zinc salt precursor. The experiments showed that the MO (with very low concentration − 5·10^−5^M) was completely decomposed after 6 hours. The PL at 360 nm excitation fixed wavelength analysis of the solid samples was correlated with their morphology.

The presence of Li atoms led to a photocatalytic activity of the doped ZnO similar to the undoped ZnO obtained from zinc acetate precursor with higher concentration. This study represents a good premise to obtain less expensive photocatalysts using carbon from biomass wastes on one hand, and optimum concentrations of doping agent for the same photocatalytic activity as for undoped materials on the other.

## Materials and Methods

### Materials

Cherry pits were purchased form local market washed with distilled water to remove impurities and dried before the pyrolysis process. Zinc salt precursors, Zn(CH_3_COO)_2_ ·2H_2_O (Sigma-Aldrich), ZnCl_2_ (Sigma-Aldrich)_,_ Zn(NO_3_)_2_ ·6H_2_O (Sigma-Aldrich) and Li_2_CO_3_ (Sigma-Aldrich) were used without further purification.

### Methods

#### Pyrolysis of cherry pits biomass for biochar synthesis

The dried cherry pits were pyrolyzed using a laboratory set-up build in Mass Transfer Laboratory of University Politehnica of Bucharest which was described in a previous study^57^. The cherry pits were introduced in a quartz column of 50 cm height and 5 cm diameter and heated by an electric resistance to 800 °C to decompose the waste biomass. The flow of CO_2_ upflowed the column while volatile compounds were evacuated and cooled in a condenser. The yield of carbon (wt. %) obtained after pyrolysis process was 24.5 ± 0.1 g.

#### Activation of carbon

Using the same set-up, the activation of the pyrolyzed cherry pits was carried out for 1 hour at 1200 °C using CO_2_ as flow gas.

#### Synthesis of carbon-ZnO and carbon-ZnO-Li_2_O nanocomposites

In a typical reaction 1,1 g of grinded biochar (inactivated or activated) was impregnated with aqueous zinc salt precursor solution at room temperature (as presented in Table 1), followed by the evaporation of the water phase. The black powder was further calcinated for 4 h at 500 °C. At the end of the calcination process the white-gray powder (0.4 g) was used for photocatalytic degradation of MO.

For the doping procedure, different amounts of Li_2_CO_3_ were used to impregnate the inactivated biochar simultaneously with Zn(CH_3_COO)_2_ (see Table 1). Thus, 0.075, 0.15, respectively 0.3 g of Li_2_CO_3_ along with 3 g of Zn(CH_3_COO)_2_ were added to the inactivated biochar. The black aliquots were treated similarly as the previous undoped samples.

## Supplementary information

Supplementary Info
