# Supplementary material for: New carbon/ZnO/Li2O nanocomposites with enhanced photocatalytic activity
Source: Sci Rep. 2019 Nov 14;9:16840. doi: 10.1038/s41598-019-53335-7 (PMC6856305; doi:10.1038/s41598-019-53335-7)
Supplement: Supplementary file 1 — Supplementary Info [file 41598_2019_53335_MOESM1_ESM.docx]

**New carbon/ZnO/Li_2_O nanocomposites with enhanced photocatalytic activity**

*(supplementary information)*

Aurel Diacon^1^, Alexandra Mocanu^1^*, Cristian Eugen Răducanu^1^, Cristina Busuioc^1^,

Raluca Șomoghi^2^, Bogdan Trică^2^, Adrian Dinescu^3^, Edina Rusen^1^

*^1^University POLITEHNICA of Bucharest, Faculty of Applied Chemistry and Materials Science,*

*Gh. Polizu Street 1-7, Bucharest, Romania, postal code 011061*

*^2^National Research and Development Institute for Chemistry and Petrochemistry – ICECHIM,*

*202 Splaiul Independenţei, Bucharest, 060021, Romania*

*^3^National Institute for Research and Development in Microtechnologies - IMT-Bucharest, 126 A, Erou Iancu Nicolae Street, PO-BOX 38-160, 023573, Bucharest, 077190, Romania*

*e-mail corresponding author:* [*alexandra.mocanu@upb.ro*](mailto:alexandra.mocanu@upb.ro)

**Figure S1.** UV-Vis reflectance measurements for carbon-ZnO,

respectively carbon-ZnO-Li_2_O photocatalysts

**Figure S2.** Tauc plots for carbon-ZnO,

respectively carbon-ZnO-Li_2_O photocatalysts

**Table S1.** *Specific surface area of the composites*

| **Sample code** | **Specific surface area (m^2^/g)** |
| --- | --- |
| CN-ZnO-3 | 10.88 |
| CN-ZnO-Li_2_O-0.075 | 4.35 |
| CN-ZnO-Li_2_O-0.15 | 5.78 |
| CN-ZnO-Li_2_O-0.3 | 5.89 |
